# Efficacy of Oral Administration of *Allium sativum* Powder “Garlic Extract” on Lipid Profile, Inflammation, and Cardiovascular Indices among Hemodialysis Patients

**DOI:** 10.1155/2021/6667453

**Published:** 2021-05-17

**Authors:** Masoumeh Asgharpour, Armin Khavandegar, Parastoo Balaei, Noora Enayati, Parham Mardi, Amirhesam Alirezaei, Mahmood Bakhtiyari

**Affiliations:** ^1^Department of Nephrology, Rouhani Hospital, Babol University of Medical Sciences, Babol, Iran; ^2^Student Research Committee, Alborz University of Medical Sciences, Karaj, Iran; ^3^Clinical Research and Development Center, Shahid Modarres Hospital, Department of Nephrology, Shahid Beheshti University of Medical Sciences, Tehran, Iran; ^4^Department of Phytochemistry, Medicinal Plants and Drug Research Institute, Shahid Beheshti University, Tehran, Iran; ^5^Non-Communicable Diseases Research Center, Alborz University of Medical Sciences, Karaj, Iran

## Abstract

**Background:**

Inflammation plays a prominent role in the deteriorating cardiovascular risk of hemodialysis patients. Utilization of herbal remedies, especially garlic extract, in these patients has resulted in promising improvement in lipid profile, inflammation, and cardiovascular markers.

**Purpose:**

In this survey, we aimed to determine the effects of oral administration of *Allium sativum* powder on lipid profile, inflammation, and cardiovascular markers among hemodialysis patients.

**Methods:**

In this interventional double-blinded randomized crossover study, 70 patients were enrolled and assigned in two groups. Each group received 300 mg of garlic powder BID or placebo for eight weeks; after a six-week wash-out period, the agents were switched between two groups so that the group who received garlic powder in the first eight weeks received placebo for the second eight weeks, and vice versa. Venous blood samples were obtained in baseline, wash-out period, and at the end of study. Following obtaining laboratory data, BUN, creatinine, Na, K, Ca, Ph, homocysteine, and lipid profile were compared.

**Results:**

No significant differences were shown at baselines of outcomes between the garlic and placebo group (*p* value>0.05). However, there was a significant decrease in absolute values of OXLDL (mean placebo = 979.63, mean garlic = 676.54; *p* value<0.001) and HCY (mean placebo = 36.54, mean garlic = 27.78; *p* value<0.001). In addition, significant treatment differences were seen in change values of Ca (placebo = 1.17, garlic = 0.21; *p* value = 0.006), TG (placebo = −6.71, garlic = -24.14; *p* value = 0.014), OXLDL (placebo = −281.30, garlic = -699.78; *p* value<0.001), and HCY (placebo = −0.24, garlic = −6.68; *p* value<0.001).

**Conclusion:**

In this study, *Allium sativum* powder demonstrated efficacy in lipid profile improvement and renal protective effects among hemodialysis patients.

## 1. Introduction

End-Stage Renal Disease (ESRD) is a vast global concern that affects individuals as well as communities worldwide [1]. Various meta-analysis predicted CKD prevalence by 11% to 13.4%, which is even higher in low and middle outcome countries [2]. Dialysis patients experience higher physical disability and have higher rates of mortality [3]. Cardiovascular disease (CVD) is the most prevalent cause of death in hemodialysis patients in most countries [4].

Classic CVD risk factors in dialysis patients are prior CVD, higher levels of C-reactive protein (CRP), left ventricular hypertrophy, diabetes, and advanced age [5]. Uremic dyslipidemia, which is a risk factor of atherosclerosis, is characterized by higher levels of triglycerides and lower levels of HDL, while changes of LDL and total cholesterol are less implied [6, 7]. Although significant improvements were seen in the medical treatment of the dialysis patient during past decades, CVD risk has remained unacceptably high and is comparable to many malignancies [8]. Studies imply that conventional medical treatments are only modifying traditional CVD risk factors where non-traditional CVD risk factors such as protein-energy malnutrition and persistent inflammation are less focused [9].

Chronic inflammation consequences in hemodialysis patients are not only due to directly increasing CVD mortality risk but also inflammation's critical role in malnutrition, chronic resistant anemia, cachexia, and bone-mineral disease, altogether result in accelerating atherosclerosis and increasing CVD death [10].

During past decades, a growing interest in the utilization of herbal remedies is seen to modify chronic diseases [11–13]. As a well-known valuable food material, garlic (*Allium sativum*) is of those herbal remedies whose usage in modifying various types of disorders has been accepted for centuries [14]. Garlic (*Allium sativum*) extract utilization as fresh garlic, standardized garlic extract, or AGE (aged garlic extract) is generally safe and well-tolerated in conventional doses [15, 16]. Garlic has shown some promises on improving hyperlipidemia [17, 18], inflammation [19], and general metabolic states, especially in chronic diseases [20, 21].

It is believed that anti-inflammatory effects of *Allium sativum*, known as garlic extract, are due to its potential to decrease cytokine production in endothelial cells, modifying adipocyte metabolic profile, and stimulating anti-inflammatory gene expression [22, 23].

Hence, we designed this study to assess the efficacy and safety of the garlic extract in reducing inflammatory status, improving lipid profile, and modifying cardiovascular risk factors in hemodialysis patients.

## 2. Materials and Methods

### 2.1. Subjects

In this double-blind, randomized clinical trial, conducted as a crossover study, 70 hemodialysis patients at Shahid Beheshti Hospital of Babol and Modarres Hospital of Tehran in 2018 were included. Subjects were eligible if they were hemodialysis patients within the age range of 18 to 70, on maintenance hemodialysis for more than six months but less than five years, and dialyzed by arteriovenous graft access. Patients suspected to have a bleeding disorder and those who took warfarin, undergoing an ileal bypass, and consumed alcohol were excluded from the study. Our study considered precisely the ethical principles of the World Medical Association Declaration of Helsinki and validated by the Ethical Committee of Research of University of Medical Sciences (IR.SBMU.RETECH.REC.1396.548) and registered in IRCT (IRCT20170725035305N2).

### 2.2. Random Allocation

**Figure 1:**
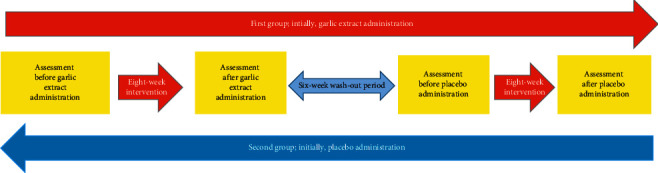
The workflow of this study. As demonstrated, each group initially received the garlic extract or placebo drug for eight weeks. After a washing-out period of six weeks, the agents were switched between groups.

In this study, we applied balanced block randomization with a block size of four; then, we used computer-generated random numbers to allocate the sequences. STATA software generated random numbers chains, consisting of one to six until the desired sample size was acquired. Given that the total number of cases to fit two people in 4 blocks is six modes, if the generated number exceeded six, the next number was regenerated, regardless of the previous number. Drugs containing garlic or placebo were designed with the same appearance, each numbered with a code in sealed envelopes. Daru Pajooh Jaber Company, Tabriz, Iran, supplied treatment drugs and placebos. Each coded number was labeled by a number from 1 to 70. The patients were randomly allocated into two even experimental groups; the first group initially received garlic extract, while the second group initially received a placebo. Both groups were identical in terms of characteristics and comorbid conditions; the participants were divided sequentially (Figure 1).

### 2.3. Study Design

During the first eight weeks of the study, the first group received 300 milligrams of standardized garlic powder (containing 1.3 milligrams of the garlic extract) BID, where the other group took a placebo. After a 6-week wash-out period, for the second 8 weeks of study, the agents were switched between groups so that the first group received a placebo, and the second group was on standardized garlic powder. All the subjects continued to take their conventional medicine during the study. Renal function tests, sodium, potassium, and lipid profile were obtained before and after every eight weeks of the study. Results were compared as garlic arm (before and after receiving garlic for eight weeks in both groups) and placebo arm of the study.

### 2.4. Sample Size

Since this is the first study determined to show garlic effects on inflammatory markers and lipid profile in hemodialysis patients, according to the optimal sample size estimation for a pilot randomized trial approach [24], a sample size of 70 precipitants is sufficient enough to detect a clinically significant effect size of 35% between groups, using a two-sided *Z*-test of the difference between proportions with 90% power and a 5% significance level.

### 2.5. Biochemical Assays and Laboratory Measurements

Venous blood samples were obtained from each participant after an overnight fast, before and after both eight weeks' phase of the study. Samples were collected into standard simple plain tubes and allowed to stand at room temperature for 20 minutes to clot and then centrifuged after 10 minutes. Biochemical parameters were done on samples (total cholesterol, triglyceride, LDL, HDL, BUN, and creatinine). Level of high sensitivity C-reactive protein (hs-CRP) was studied with the usage of latex immunoassay method, and the results were expressed as mg/l. Erythrocyte sedimentation rate (ESR) was measured by the Wintrobe method, and the results were expressed as millimeters in one hour.

### 2.6. Statistical Analysis

Departure from normality assumption was assessed by the Kolmogorov–Smirnov test. Mean ± standard deviation (SD) was used in order to present the data. For each variable, differences of values were calculated before and after each 8-week treatment of garlic powder or placebo and compared between the two arms of the study (garlic arm comparing values before and after garlic intake in both groups, and placebo arm comparing effects of placebo in both groups) using analysis of covariance (ANCOVA). We compared baseline values of biochemical markers with their posttreatment values in each arm separately by paired *t*-test or Wilcoxon signed-rank test. A *p* value below 0.05 was considered significant in all analyses. All statistical analyses were accomplished using the SPSS software version 22.

## 3. Results

Seventy subjects (38 males and 32 females) entered the trials and completed the study. Thirty-five subjects (23 males and 12 females) were randomized to receive the allium sativum powder tablets as the drug and another thirty-five subjects (15 males and 20 females) to receive placebo tablets for the eight weeks in the first period. Homogeneity of sex and sequence (drug-placebo and placebo-drug) was observed in both periods (*p* value>0.05).

Baseline value, absolute values, and changes after eight weeks were expressed for all outcomes. According to the results, no significant differences were shown at baselines of outcomes between the garlic and placebo group (*p* value>0.05). However, there was a significant decrease in absolute values of OXLDL (mean placebo =  979.63, mean garlic = 676.54; *p* value<0.001) and HCY (mean placebo = 36.54, mean garlic = 27.78; *p* value<0.001). In addition, significant treatment differences were seen in change values of Ca (placebo = 1.17, garlic = 0.21; *p* value = 0.006), TG (placebo = −6.71, garlic = -24.14; *p* value = 0.014), OXLDL (placebo = -281.30, garlic = −699.78; *p* value<0.001), and HCY (placebo = −0.24, garlic = -6.68; *p* value<0.001) (Table 1). Moreover, Table 1 shows the subjects' mean outcomes including Cr, TG, OXLDL, and HCY, before and after, for patients who received garlic and placebo.

Table 2 shows the baseline effect, carryover effect, period effect, and main effect of treatment. There was a significant positive change in Cr (coefficient estimation of garlic vs. placebo = 0.58; *p* value = 0.047) between garlic and placebo. Accordingly, the mean Cr for subjects who received garlic was 0.53 unit higher than those who received placebo controlling baseline effect, carryover effect, and period effect. Furthermore, the significant negative change was estimated in TG (coefficient estimation of garlic vs. placebo = −16.21; *p* value = 0.037), OXLDL (coefficient estimation of garlic vs. placebo = −327.57; *p* value<0.001), and HCY (coefficient estimation of garlic vs. placebo = −7.29; *p* value<0.001). Based on the results, mean TG, OXLDL, and HCY in the garlic group were 16.21, 325.27, and 7.29 units lower than in the placebo group controlling other effects including baseline, carryover, and period.

## 4. Discussion

Our survey was a double-blind placebo-controlled crossover trial that was implanted to demonstrate the effect of the garlic extract on some of the inflammatory markers, lipid profile, and renal function tests.

This study mainly showed that garlic might be useful in reducing the lipid profile in hemodialysis patients. We observed that the levels of TAG and OXLDL significantly decreased in the garlic arm of the study, while there was no significant decrease in cholesterol level compared to the placebo group. Ried et al.'s clinical trial in 2016 demonstrated beneficial effects of the garlic extract on blood pressure in uncontrolled hypertensive patients [25]. However, this study could not show any significant changes in other cardiovascular biomarkers, including total cholesterol and LDL, presumably due to insufficient sample size. Our study similarly revealed no significant changes in cholesterol level but reversely showed a remarkable decrease in OXLDL level in garlic arm of the study.

Another study by Pevez-Torres et al. in 2016 showed that cholesterol levels were reduced by using AGE (aged garlic extract) in metabolic syndrome rats [26]. In contrast, the cholesterol levels in the garlic arm of the present study did not decrease more significantly compared to the placebo arm. In a study by Zhang et al. performing various pharmacological and molecular analyses on garlic, they eventually claimed that fermented black garlic could potentially protect the body against cardiovascular diseases [27].

In a study of Mohammadi et al. in 2014, treatment with the garlic extract significantly decreased total cholesterol, LDL, and triglycerides in hypercholesterolemic mice [28], which is compatible with the results of the present study. A clinical trial by Kojuri et al. in 2007 also demonstrated the beneficial effects of the garlic extract on cholesterol, LDL, and HDL [29]. Similarly, the changes in lipid profiles, except cholesterol, in our study were significant. A known effect of this extract is its ability to decrease the cholesterol level [30]. Particular sample group of patients (hemodialysis patients) and their basal cholesterol level justifies the absence of no significant decrease in cholesterol level. Another review article performed by Yeh et al. in 2001 demonstrated the cholesterol-lowering effects of the garlic extract [31]. The present study does not support these findings.

In a double-blinded placebo-control study in 2019, comparing the effects of 2400 mg daily administration of the aged garlic extract (AGE) with a placebo group, they postulated that a 12-month administration of AGE supplement might preserve and even improve the microcirculation; probably due to the inhibition of the atherosclerosis process and affecting endothelial function [32].

In another study performed by Sobenin et al., a review of the potential anti-atherosclerotic mechanism, by which garlic can probably modify the lipid extract, has been provided; at last, they claimed that although evidence is not sufficient enough, usage of garlic preparation as a complement has been recommended [33]. In another systematic review, Shabani et al. articulated that garlic can decrease lipid profile besides glucose parameters. They admitted that garlic is potentially useful in patients suffering from cardiovascular diseases and diabetes [34].

Elevated plasma homocysteine was previously known to contributed to increased cardiovascular disorders risk [35–37]. In an animal study performed in 2006, it was postulated that, besides cholesterol-lowering effect, blood pressure lowering potential, and antioxidant entity of garlic, it could also decrease cardiovascular disorders risk by lowering plasma homocysteine [38]. Comparably, in this study, there was a significant difference in lowering effect of plasma homocysteine in placebo and garlic arms of the study.

In an animal study performed by Oladele et al. in 2020, it was believed that garlic has a renal protective effect [39]. In a famous study, implemented by El-Demerdash in 2005, it was concluded that garlic has an alleviative effect on rats' kidney damage caused by diabetes [40]. Comparably, in our study, creatinine was significantly lowered in garlic arm of the study.

In a study by Zare et al. in 2019, performing a parallel double-blinded study, to compare the effect of the garlic extract and placebo in 42 peritoneal dialysis patients, it was revealed that 400 mg of the standardized garlic extract twice a day for 8 weeks resulted in remarkable reduction in inflammatory biomarkers [41].

A review article by Arreola et al. in 2015 on anti-inflammatory effects of garlic compounds showed that garlic enhanced the immune system by stimulating specific cells such as macrophages, lymphocytes, natural killer (NK) cells, dendritic cells, and eosinophils by various mechanisms [42]. The results of the present study also demonstrate a significant decrease in inflammatory biomarkers of ESR and CRP by using the garlic extract.

In a recent clinical trial in 2019, Zare et al. showed that 400 milligrams of the standardized garlic extract twice a day for eight weeks resulted in a significant reduction in IL-6, CRP, and ESR [41]. In the present study, likewise, significant changes of ESR and CRP were seen in the garlic arm.

In this study, we eventually postulated that the garlic extract is useful in decreasing TAG, OXLDL, and homocysteine level. Besides, in the garlic arm of this study, calcium and creatinine were remarkably lower in the placebo group. Regarding other laboratory ingredients, consisting of potassium, BUN, cholesterol, sodium, and ANT, there were not any significant differences between placebo and garlic groups. As a limitation, it should be mentioned that small sizes of investigational samples may be insufficient to indicate the effects of garlic on various biomarkers. Hence, conducting further trials with larger sample sizes is recommended.

## 5. Conclusion

Concerning the significant role of inflammation and dyslipidemia in cardiovascular mortality risk factors of hemodialysis patients, we suggest the use of garlic in selected patients. However, more extensive clinical trials are probably needed to assess this agent's safety and efficacy.

## Figures and Tables

**Table 1:** Effect of *Allium sativum* powder tablets vs. placebo tablets on lipid profile, inflammation, and cardiovascular indexes.

		Garlic (*n* = 70)		Placebo (*n* = 70)		Statistical significance of the effect: *P* (garlic vs. placebo)
		Mean	Std dev	Mean	Std dev	
k	Before	5.49	0.67	5.63	0.78	0.247
	After	5.49	0.57	5.57	0.62	0.418
	Change	0.00	0.70	−0.07	0.74	0.617
BUN	Before	70.99	20.99	71.76	17.61	0.814
	After	70.20	19.26	70.16	20.43	0.990
	Change	−0.79	16.46	−1.60	15.89	0.766

Cr	Before	11.05	4.00	12.44	11.89	0.357
	After	10.61	3.45	10.10	3.52	0.393
	Change	−0.28	3.12	−2.34	11.43	0.148

Ca	Before	7.86	0.69	7.86	0.71	0.973
	After	8.19	0.72	8.24	0.92	0.712
	Change	0.21	1.36	1.17	2.50	0.006

Ph	Before	6.10	1.34	6.00	1.27	0.660
	After	5.64	1.31	5.87	1.26	0.304
	Change	−0.46	1.17	−0.14	0.99	0.082

Chol	Before	144.26	31.17	140.44	35.47	0.500
	After	138.51	32.20	137.61	37.41	0.879
	Change	−5.74	20.58	−2.83	14.29	0.332

TG	Before	162.51	96.24	152.56	89.95	0.528
	After	138.37	87.25	145.84	97.21	0.633
	Change	−24.14	30.33	−6.71	49.98	0.014

Na	Before	139.96	6.18	141.04	6.68	0.320
	After	137.59	3.15	138.31	3.48	0.196
	Change	−2.37	6.46	−2.73	7.47	0.763

OXLDL	Before	1376.31	715.92	1260.92	658.17	0.323
	After	676.54	258.93	979.63	408.24	<0.001
	Change	−699.78	685.84	−281.30	594.52	<0.001

TAC	Before	0.92	0.67	0.94	0.66	0.848
	After	1.04	0.70	0.85	0.54	0.068
	Change	0.12	0.74	−0.10	0.64	0.066

HCY	Before	34.46	11.11	36.77	12.72	0.254
	After	27.78	9.79	36.54	11.38	<0.001
	change	−6.68	9.38	−0.24	8.50	<0.001

k: kalium; BUN: blood urea nitrogen; Cr: creatinine; Ca: calcium; Ph: the power of hydrogen; Chol: cholesterol; TG: triglyceride; Na: natrium; OXLDL: oxidized low-density lipoprotein; TAC: total antioxidant capacity; HCY: homocysteine.

**Table 2:** Randomized crossover, double-blind, and placebo-controlled trials investigating the oral effect administration of *Allium sativum* powder on lipid profile, inflammation, and cardiovascular indexes.

	Baseline effect			Carryover effect: drug-placebo vs. placebo-drug			Period effect: period 1 vs. period 2			Treatment effect: drug vs. placebo		
	Est. (SE)	df	*p*	Est.(SE)	df	*p*	Est.(SE)	df	*p*	Est.(SE)	df	*p*
K	0.30 (0.06)	67	<0.001	−0.08 (0.10)	68	0.429	−0.06 (0.08)	67	0.479	−0.04 (0.08)	67	0.630
BUN	0.62 (0.06)	67	<0.001	−5.07 (2.44)	68	0.041	11.32 (2.22)	67	<0.001	0.52 (2.21)	67	0.814
Cr	0.05 (0.02)	66	0.018	−0.79 (0.75)	68	0.302	0.33 (0.28)	66	0.248	0.58 (0.28)	66	0.047
Ca	0.55 (0.08)	58	<0.001	0.19 (0.13)	68	0.134	−0.60 (0.08)	58	<0.001	0.02 (0.08)	58	0.857
Ph	0.74 (0.06)	67	<0.001	−0.43 (0.15)	68	0.007	−0.57 (0.17)	68	0.001	−0.30 (0.16)	67	0.068
Chol	0.93 (0.04)	67	<0.001	−4.44 (2.52)	68	0.082	−16.28 (2.72)	67	<0.001	−2.66 (2.71)	67	0.329
TG	0.88 (0.03)	67	<0.001	1.01 (5.44)	68	0.864	12.17 (7.62)	67	0.115	−16.21 (7.60)	67	0.037
Na	0.06 (0.06)	67	0.306	0.36 (0.64)	68	0.573	−0.26 (0.68)	67	0.708	−0.66 (0.49)	67	0.177
OXLDL	0.19 (0.05)	67	<0.001	−36.96 (61.83)	68	0.552	−66.85 (57.14)	67	0.246	−325.27 (44.35)	67	<0.001
TAC	0.49 (0.06)	67	<0.001	−0.15 (0.07)	68	0.043	0.09 (0.12)	67	0.461	0.21 (0.11)	66	0.077
HCY	0.63 (0.05)	67	<0.001	1.94 (1.29)	68	0.135	−2.05 (1.32)	67	0.127	−7.29 (1.33)	67	<0.001

k: kalium; BUN: blood urea nitrogen; Cr: creatinine; Ca: calcium; Ph: the power of hydrogen; Chol: cholesterol; TG: triglyceride; Na: natrium; OXLDL: oxidized low-density lipoprotein; TAC: total antioxidant capacity; HCY: homocysteine.

## Data Availability

The datasets used and analyzed during the current study are available from the corresponding author upon reasonable request.
